# Single-Walled Carbon Nanotube Sensor Selection for the Detection of MicroRNA Biomarkers for Acute Myocardial Infarction as a Case Study

**DOI:** 10.1021/acssensors.3c00633

**Published:** 2023-09-13

**Authors:** Adi Hendler-Neumark, Verena Wulf, Gili Bisker

**Affiliations:** † Department of Biomedical Engineering, Faculty of Engineering, Tel Aviv University, Tel Aviv 6997801, Israel; ‡ Center for Physics and Chemistry of Living Systems, Tel-Aviv University, Tel Aviv 6997801, Israel; § Center for Nanoscience and Nanotechnology, Tel-Aviv University, Tel Aviv 6997801, Israel; ∥ Center for Light-Matter Interaction, Tel-Aviv University, Tel Aviv 6997801, Israel

**Keywords:** single-walled carbon nanotubes, microRNA, acute myocardial infarction, fluorescence, biosensor

## Abstract

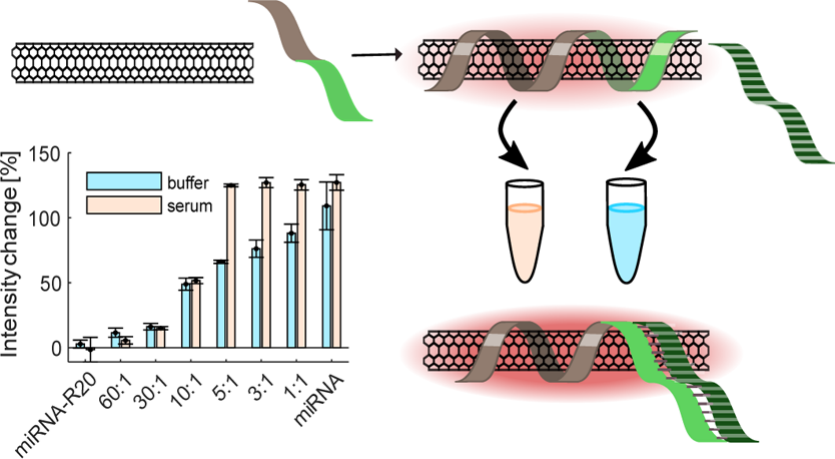

MicroRNAs (miRNAs) are single-stranded non-coding short ribonucleic acid sequences that take part in many cellular and biological processes. Recent studies have shown that altered expression of miRNAs is involved in pathological processes, and they can thus be considered biomarkers for the early detection of various diseases. Here, we demonstrate a selection and elimination process of fluorescent single-walled carbon nanotube (SWCNT) sensors for miRNA biomarkers based on RNA-DNA hybridization with a complementary DNA recognition unit bound to the SWCNT surface. We use known miRNA biomarkers for acute myocardial infarction (AMI), commonly known as a heart attack, as a case study. We have selected five possible miRNA biomarkers which are selective and specific to AMI and tested DNA-SWCNT sensor candidates with the target DNA and RNA sequences in different environments. Out of these five miRNA sensors, three could recognize the complementary DNA or RNA sequence in a buffer, showing fluorescence modulation of the SWCNT in response to the target sequence. Out of the three working sensors in buffer, only one could function in serum and was selected for further testing. The chosen sensor, SWCNT-miDNA208a, showed high specificity and selectivity toward the target sequence, with better performance in serum compared to a buffer environment. The SWCNT sensor selection pipeline highlights the importance of testing sensor candidates in the appropriate environment and can be extended to other libraries of biomarkers.

MicroRNAs (miRNAs) are single-stranded, non-coding, short ribonucleic acid sequences that take part in many cellular and biological processes, e.g., cell migration, secretion, apoptosis, aging, proliferation, and differentiation, by altering the target protein levels.^1^ Altered expression of various miRNAs is involved in pathological processes, which makes them suitable biomarkers for the early detection of diseases.^2^

The current standard to measure miRNA concentrations is via quantitative PCR, which requires purification and amplification of the sample, which are time-consuming and insert variability. Other methods, such as microarray, do not require amplification but show lower sensitivity with high rates of false-positive results.^3−5^ Additional strategies that avoid labeling, amplification, and purification from biofluids include nanoparticle-based detection,^6^ ligase chain reaction,^7^ or rolling-circle amplification.^8^ Optical miRNA-detection with photoresponsive nanomaterials, e.g., gold nanoparticles,^6^ has many advantages, among them are the immediate read-out, the requirement of small sample volumes, and the photostability of the sensors compared to organic fluorescent dyes.^9^ Several studies have shown successful miRNA detection with fluorescent nanoparticles,^6,10^ among them single-walled carbon nanotubes (SWCNTs) have been shown to detect miRNA targets in vivo.^11^

SWCNTs can be described as graphene sheets rolled up into tubes, where the roll-up vector not only determines the diameter but also the chemical, physical, and optical properties of the resulting SWCNT chirality. Semiconducting SWCNTs show fluorescence emission in the near-infrared (NIR) spectral range, which overlaps with the transparency window of biological tissue,^12−15^ rendering them excellent optical sensors for biomedical sensing and imaging applications, even in vivo.^16−19^ As a further advantage, SWCNTs do not suffer from photobleaching or blinking, and they show high biocompatibility.^13,19−21^ The sensing ability of SWCNTs is based on fluorescence emission intensity changes or emission wavelength shifts, induced through changes in the dielectric environment in close proximity to the SWCNT-surface, e.g., via analyte binding to the SWCNT functionalization,^13,22^ direct modulation of the corona phase,^23−25^ or rearrangement of the surrounding molecules on the SWCNT surface.^26−28^ Synthetic surface functionalization for SWCNTs, e.g., polymers or single-stranded DNA, being nonspecific to an analyte by themselves, have been shown to form a three-dimensional corona phase around the SWCNT surface that can mediate the interaction with the SWCNT environment or the binding of specific analyte molecules.^29−39^ Nevertheless, especially in crowded biological environments, like serum, more specific recognition sites, e.g., based on miRNA-DNA hybridization, might be of advantage.^40,41^ Using transmission electron microscopy,^42,43^ atomic force microscopy,^44,45^ or small-angle X-ray scattering,^46^ several studies have demonstrated an order organization of the DNA strand around the nanotube scaffold attributed to π–π stacking interaction between the nucleotides and the SWCNT surface. Optical nanosensors, whose analyte recognition is based on DNA hybridization, are equipped with a DNA strand complementary to the analyte, but the final analyte binding mechanism can still face multiple obstacles. For example, the recognition DNA strand, which is expected to bind a miRNA target, might have a higher affinity to the nanosensor surface, hindering the hybridization.^11,47,48^

In this study, we demonstrate a method for selecting and eliminating sensor candidates for acute myocardial infarction (AMI) as a case study. AMI, commonly known as heart attack, is one of the major causes of death worldwide.^49−51^ Patients suffering from heart attacks can get better life-saving treatments when promptly diagnosed after the onset of the symptoms. The conventional AMI “gold standard” biomarker is an elevated level of cardiac troponins. However, the rise in troponins is delayed by approximately 4–6 h after the onset of the AMI symptoms, and the peak troponin levels are reached only after 18–24 hours.^52−54^ Recent studies have identified a new class of potential miRNA biomarkers for AMI, which reach peak levels within 3–12 h after the onset of the symptoms.^55−57^ We examined sensors based on the hybridization reaction of the target miRNA with DNA-suspended fluorescent SWCNTs. The sensing principle relies on the successful and specific hybridization of the miRNA with its complementary DNA in proximity to the SWCNT surface and the translation of the binding event into a fluorescence modulation of the SWCNT-miDNA sensor, detectable via NIR fluorescence spectroscopy. In the first step, the SWCNTs are suspended with a single-stranded DNA sequence consisting of an anchor unit that binds strongly to the SWCNTs and a recognition unit, complementary to known miRNA AMI-markers, i.e., miRNA29b-1, miRNA122, miRNA192, miRNA208a, and miRNA499.^49,58−61^ Single-stranded DNA is more stable than its RNA counterpart, which can suffer from spontaneous hydrolysis and degradation by ribonucleases (RNases). Therefore, we first test the sensors’ response to a complementary DNA target (miDNA) and then to the miRNA target. This elimination is carried out first in buffer and then in serum, where a more biological, crowded environment is simulated, representing the appropriate environment of the target (Scheme 1). We identified three sensors (SWCNT-miDNA208a, SWCNT-miDNA122, and SWCNT-miDNA29-1) that showed fluorescence response in buffer for their respective target analytes. Out of these three candidates, SWCNT-miDNA208a showed a fluorescence response to its target miRNA in serum and was selected as the optimal sensor from the tested library. The SWCNT-miDNA208a sensor showed a fluorescence intensity response also at the single-sensor level, demonstrating the feasibility and potential of this approach for future developments and optimization. Finally, we verified the specificity of the miRNA sensor in the presence of mutated miRNA sequences and its selectivity in the presence of random RNA sequences. We observed that the specificity and the selectivity of the SWCNT-miDNA sensor toward its miRNA target is higher in serum than that in buffer. Our selection process shows the variability in the affinity between the different sensors and their respective analytes and highlights the crucial role played by the environment on the sensor performance. Our approach can be extended to other sensor candidates and target biomarker libraries.

**Scheme 1:**
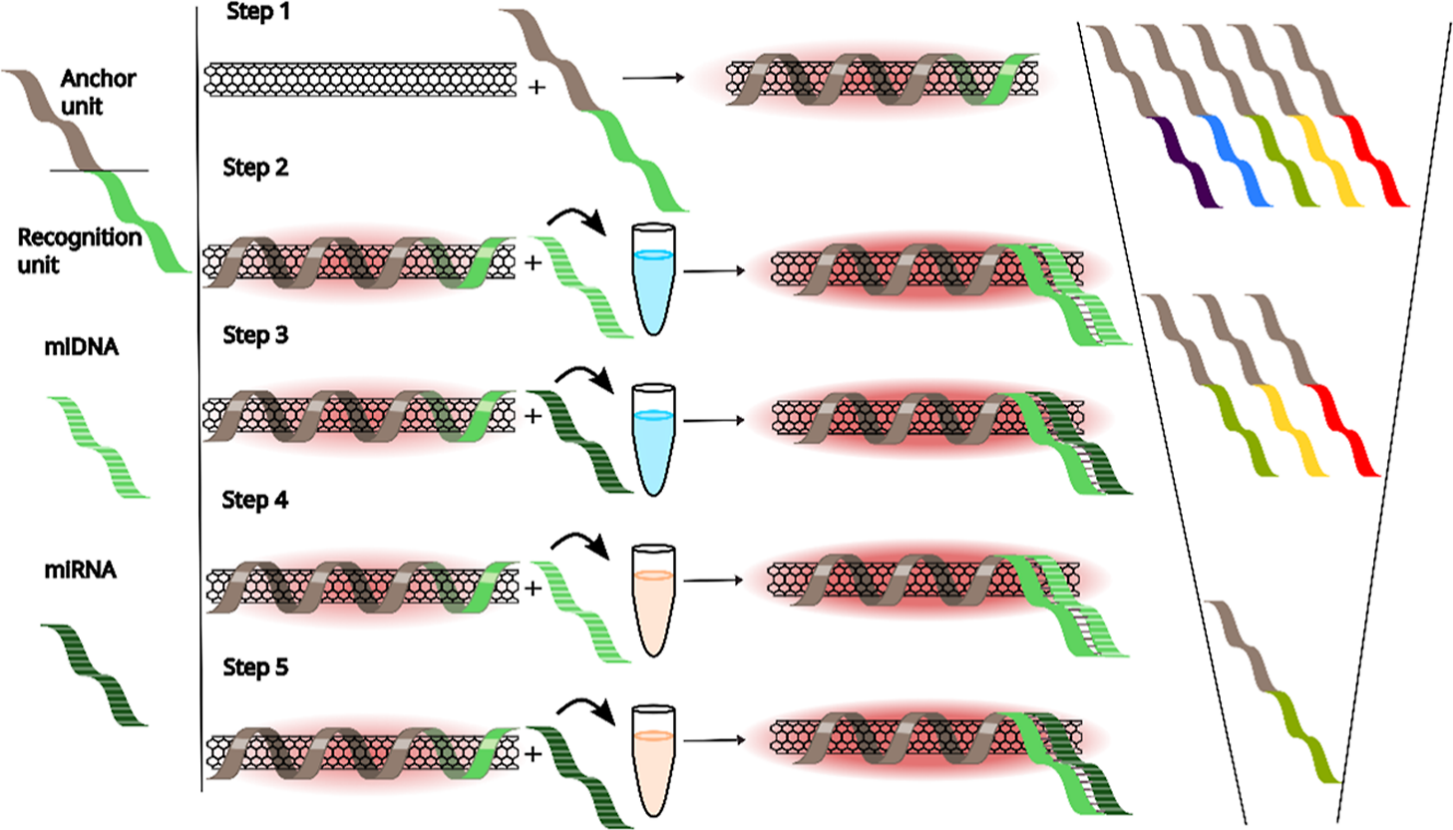
Schematic Representation of the Selection and Elimination Process for Identifying an Optical SWCNT Sensor for miRNA Step 1: The DNA used for suspending the SWCNTs contains two sections, the SWCNT-anchor unit (gray) and the miDNA recognition unit (light green, as an example). Several recognition units are tested (purple, blue, green, yellow, and red). Steps 2 and 3: All DNAs that are able to suspend SWCNTs, are first tested in buffer with their complementary miDNA (light green with stripes) and then with their complementary target miRNA (dark green with stripes). Only some of the DNA sequences used for suspending SWCNTs result in a fluorescence response to their respective targets in buffer (green, yellow, and red). Steps 4 and 5: The sensors that show fluorescence response in buffer are tested again in serum with the complementary miDNA and then with the complementary miRNA. Only one sensor showed a response in serum (green). The right side of the scheme shows the sequential selection of successful sensors with each step in the elimination process. The helical structure of the DNA around the SWCNT is used as an illustration of the corona phase formed by the DNA sequence.

## Results and Discussion

**Figure 1:**
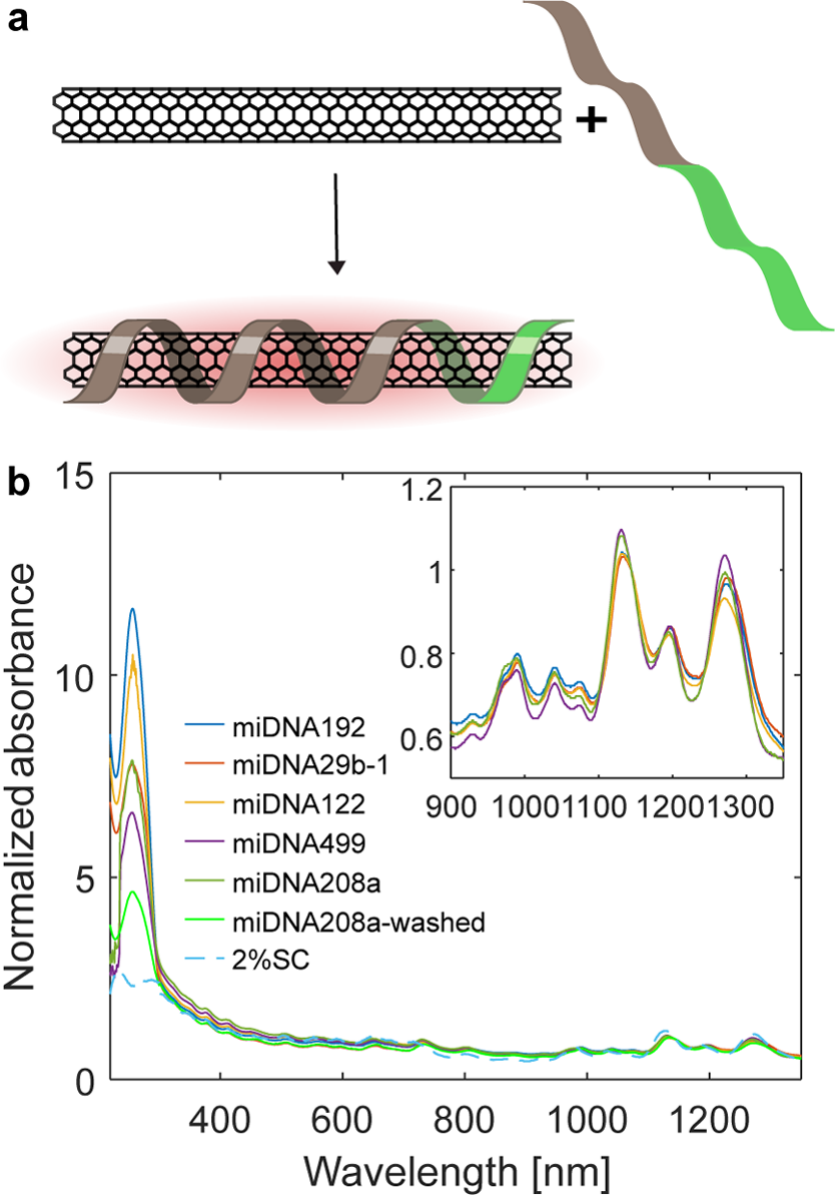
SWCNT-miDNA absorption spectra. (a) Schematic representation of the SWCNT functionalization with the DNA sequence containing an anchor unit (gray) and a miDNA recognition unit (light green). (b) Absorbance spectra of SWCNT-miDNA normalized to the peak at 1144 nm. The peak at 260 nm corresponds to the DNA in the suspension of SWCNT-miDNA192 (blue), SWCNT-miDNA29b-1 (red), SWCNT-miDNA122 (yellow), SWCNT-miDNA499 (purple), SWCNT-miDNA208a (green), SWCNT-miDNA208a from which excess DNA was filtered out (light green), and SWCNT-2% SC (light blue dashed line). Inset: NIR absorbance of the SWCNT-miDNA.

To suspend the SWCNTs and functionalize them with a recognition site for miRNA, we used DNA oligonucleotide sequences consisting of two functional regions: an anchor unit to attach non-covalently to the SWCNTs, (GT)_15_,^43,62^ and a recognition segment complementary to its respective miRNA target sequence (Figure 1a). The recognition units are complementary to five different miRNA targets, i.e., miRNA29b-1, miRNA122, miRNA192, miRNA208a, and miRNA499, that were shown previously as biomarkers for AMI.^49,58−61^ All DNA sequences were able to suspend SWCNTs, resulting in stable colloidal suspensions, as indicated by the sharp distinguishable absorption peaks of the different SWCNT-chiralities (inset is shown in Figure 1b). In the UV range of the spectra, the characteristic peak of DNA at 260 nm was clearly visible in all of our samples, even after the removal of excess miDNA from the suspension. In contrast, a control suspension of SWCNTs suspended by sodium cholate (SC) did not show absorption in the UV (Figure 1b).

### Fluorescence Response of SWCNT-miDNA Sensors to Their Target miDNA and miRNA in Buffer

**Figure 2:**
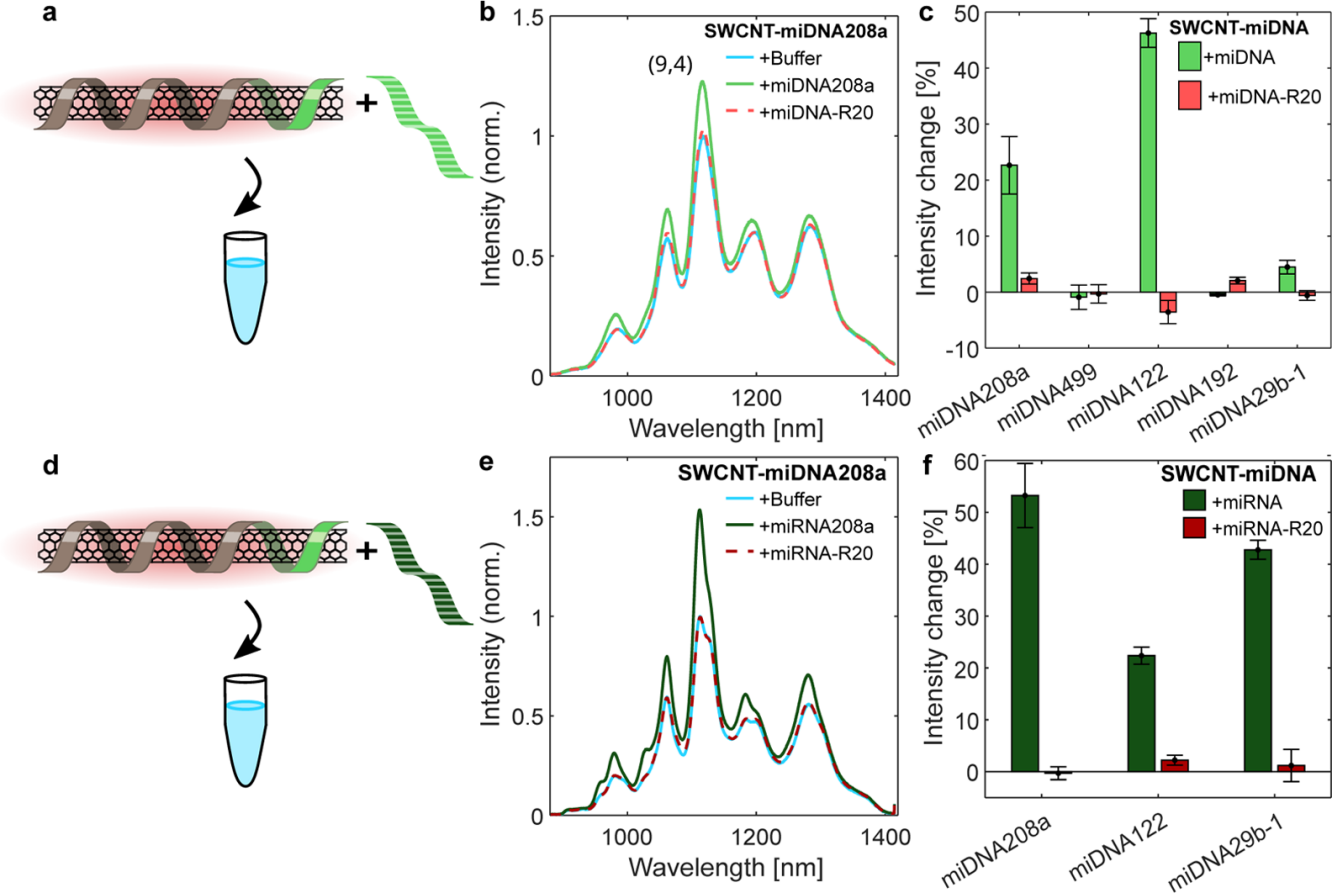
Fluorescence response of the SWCNT-miDNA sensors in buffer. (a) SWCNT-miDNA sensors with miDNA in buffer. (b) Normalized fluorescence spectra of SWCNT-miDNA208a in buffer (blue), after the addition of 10 μM miDNA208a (light green), and after the addition of 10 μM miDNA-R20 (light red), following 4 h of incubation. (c) Normalized fluorescence response of the (9.4) chirality for all SWCNT-miDNA sensors toward miDNA (light green) and miDNA-R20 (light red). (d) SWCNT-miDNA sensors with miRNA in buffer. (e) Normalized fluorescence spectra of SWCNT-miDNA208a in buffer (blue), after the addition of 10 μM miRNA208a (dark green), and after the addition of a 10 μM miRNA-R20 (dark red), following 4 h of incubation. (f) Normalized fluorescence response for all SWCNT-miDNA sensors toward miRNA (dark green) and miRNA-R20 (dark red).

Although all DNA sequences tested are able to suspend the SWCNTs, it does not necessarily mean that all recognition DNA segments can hybridize with their respective complementary target strand and translate to a fluorescence response. The recognition sequence might be tightly bound to the SWCNT surface or coiled into the corona phase, hindering hybridization. Moreover, even if successful hybridization occurs, it may not be translated to a detectable fluorescence modulation of the SWCNTs. Due to the easier handling in the laboratory and higher stability of DNA compared to RNA, we first tested the fluorescence response of our sensor candidates with the complementary miDNA (Figure 2a). These experiments were conducted in saline-sodium citrate (SSC) buffer with 0.2% w/v sodium dodecylbezenesulfonate (SDBS),^11^ based on previous findings which showed that SDBS increased the fluorescence response of SWCNT sensors.

Figure 2b shows the fluorescence intensity of the SWCNT-miDNA208a sensors after the addition of only buffer, miDNA208a, or the addition of an equal concentration of a random DNA sequence (miDNA-R20) of the same length, following 4 h of incubation. The fluorescence spectra of the other sensor candidates are shown in Figure S1. The sensors SWCNT-miDNA208a, SWCNT-miDNA122, and SWCNT-miDNA29b-1 showed a significant fluorescence intensity increase in response to the interaction with the miDNA of 22, 46, and 4.5%, respectively, which was much higher than the response to the respective random miDNA-R20 control (Figure 2c). In the case of SWCNT-miDNA122 and SWCNT-miDNA29b-1, there was a small decrease in the fluorescence intensity in response to the miDNA-R20 control, which could be due to nonspecific binding to the SWCNT surface rather than hybridization with the DNA corona. The SWCNT-miDNA192 and SWCNT-miDNA499 did not show a fluorescence response to the addition of their target miDNA, indicating that no sufficient hybridization occurred or that hybridization did not lead to a detectable fluorescence modulation. The stability of the hybridization is mostly affected by the CG content of the sequences.^63^ However, it cannot explain the variability in the responses since our target sequences, including the random control sequence, all have similar CG-content. We, therefore, assume that hybridization can be hindered by different affinities of the recognition segment of the functionalization to the SWCNT surface.^43^ As the stability of the hybridization between DNA/DNA and DNA/RNA is similar, we continued to test the sensor recognition of miRNA in buffer only with the sensors that showed fluorescence response to miDNA, namely, SWCNT-miDNA208a, SWCNT-miDNA122, and SWCNT-miDNA29b-1. The respective sensors were then evaluated with their complementary miRNA sequences or random RNA sequence (miRNA-R20) of the same length as a control (Figure 2d). Figure 2e shows the fluorescence intensity of SWCNT-miDNA208a after the addition of buffer, miRNA208a, or the addition of an equal concentration of a random RNA sequence of the same length (miRNA-R20), following 4 h of incubation. The fluorescence spectra of the other sensor candidates are shown in Figure S2. The fluorescence response of the SWCNT-miDNA208a in buffer over time is shown in Figure S3 for all SWCNT-miDNA208a samples, to which buffer, miRNA208, or miRNA-R20 were added. All three sensors, SWCNT-miDNA208a, SWCNT-miDNA122, and SWCNT-miDNA29b-1, showed a response to the miRNA target sequence of 53, 22, and 42%, respectively (Figure 2f), attributed to the hybridization with the complementary RNA recognition and the transduction to a detectable fluorescence modulation of the SWCNTs. In contrast, there was a negligible response to the random sequence (miRNA-R20) in the range of 0–2% for all three sensors.

The intensity changes of SWCNT-miDNA208a, SWCNT-miDNA122, and SWCNT-miDNA29b-1, in response to their respective miRNA targets (Figure 2f), differ from their response to the miDNA targets (Figure 2c). While the response of SWCNT-miDNA122 was higher to the miDNA compared to the miRNA, the response of SWCNT-miDNA208a and SWCNT-miDNA29b-1 was higher to their miRNA targets. Differences in the fluorescence response of the SWCNT-miDNA sensors toward miDNA and miRNA can be due to differences in the chemical composition of RNA and DNA and the structural conformation adopted by the hybridized strands on the SWCNT surface. Since the three selected sensors demonstrated a significant fluorescence response toward the AMI-markers miRNA208a, miRNA122, and miRNA29-1 in buffer, we assessed their performance in serum.

### Fluorescence Response of SWCNT-miDNA Sensors to Their Target miDNA and miRNA in Serum

**Figure 3:**
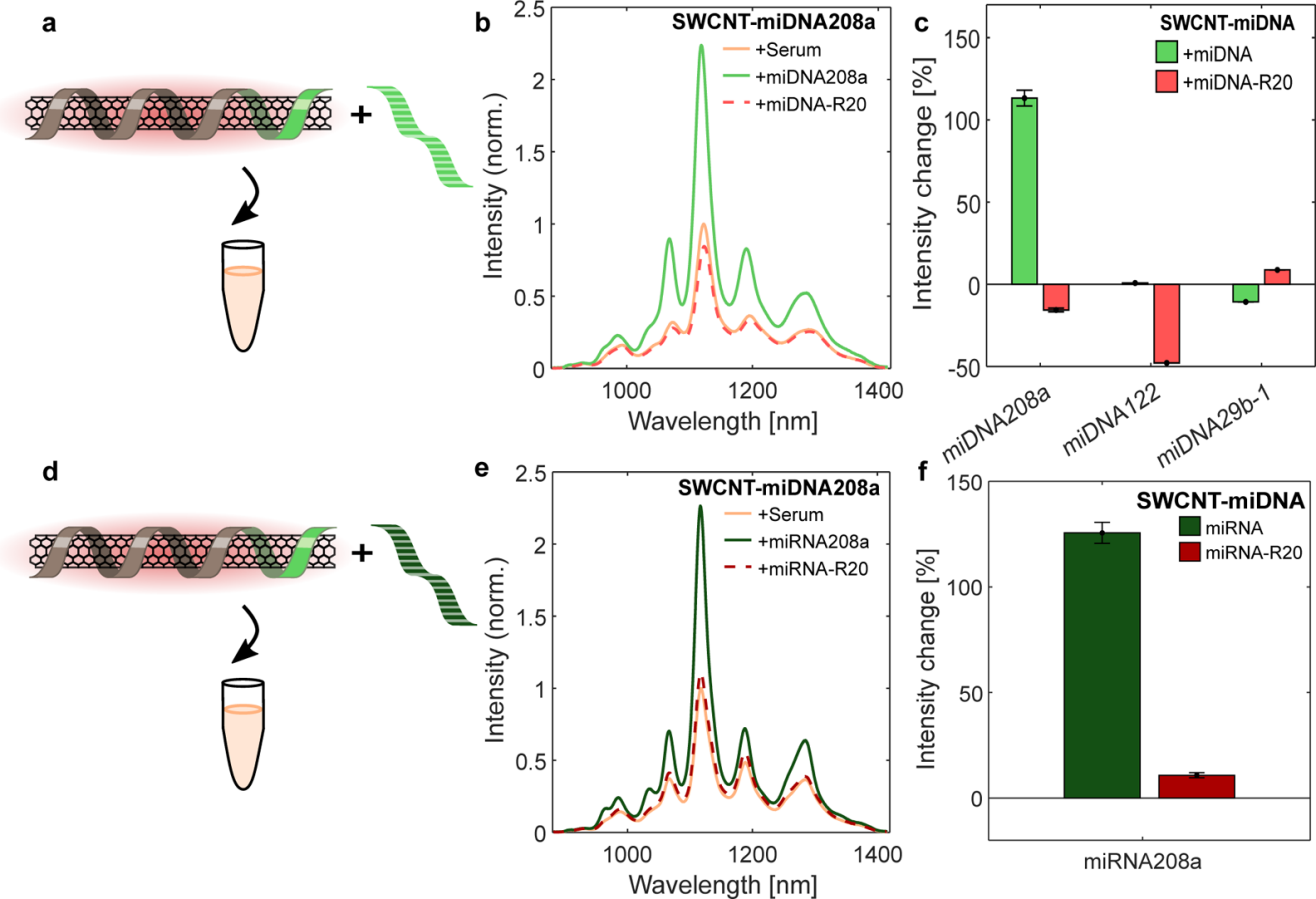
Fluorescence response of the SWCNT-miDNA sensors in serum. (a) SWCNT-miDNA sensors are exposed to their respective miDNA in serum. (b) Normalized fluorescence spectra of SWCNT-miDNA208a in serum (brown), after the addition of 10 μM miDNA208a (light green), and after the addition of a 10 μM miDNA-R20 (light red), following 4 h of incubation. (c) Intensity changes of the fluorescence response of all SWCNT-miDNA sensors toward miDNA (light green) and miDNA-R20 (light red). (d) SWCNT-miDNA sensor is exposed to their respective miRNA in serum. (e) Normalized fluorescence spectra of SWCNT-miDRNA208a in serum (brown), after the addition of 10 μM miRNA208a (dark green), and after the addition of 10 μM miRNA-R20 (dark red), following 4 h of incubation. (f) Intensity changes of the fluorescence response of SWCNT-miDNA208a sensor toward miRNA (dark green) and miRNA-R20 (dark red).

We have narrowed the miRNA pool down to three miRNAs (miRNA208a, miRNA122, and miRNA29b-1) that can be detected in buffer with our sensors. Nevertheless, we are aiming to detect miRNA in biological samples, i.e., complex environments that are crowded with proteins, enzymes, lipids, salts, and other biomolecules. To this end, we tested the sensing performance of the three SWCNT-miDNA sensors in fetal bovine serum (FBS).^64^ miDNA is not cleavable by RNases that are abundant in serum. Thus, we first measured the sensor response to miDNA (Figure 3a). We equilibrated the sensors in 10% FBS and recorded the fluorescence spectra after the addition of 10% FBS, 10 μM of the respective complementary miDNA, or miDNA-R20, following 4 h of incubation (Figures 3b and S4). Only SWCNT-miDNA208a showed a significant fluorescence response of 113% intensity increase to the complementary miDNA, whereas the control random sequence (miDNA-R20) resulted in an intensity decrease of 15% (Figure 3c). The SWCNT-miDNA122 showed no response toward the miDNA, and SWCNT-miDNA29b-1 showed an intensity decrease of 10%, which may be due to nonspecific binding of the miDNA to the SWCNTs rather than hybridization to the complementary unit. The difference in the response of the sensors toward their targets in buffer and serum might result from a complex corona phase formed around the SWCNT by components contained in the serum,^11,65^ hindering the hybridization.

Since only SWCNT-miDNA208a showed a strong fluorescence response to its target miDNA, we continued to assess its activity in serum for miRNA (Figure 3d). It was previously reported^66^ that synthetic RNA sequences can be degraded by RNases present in the serum. Therefore, we added proteinase K, a serine protease that can inactivate RNases, before the addition of the target miRNA sequence.^11,67^ The SWCNT-miDNA208a sensor was equilibrated in 10% FBS with 0.5 mg mL^–1^ proteinase K. After the addition of 10 μM miRNA208a or miRNA-R20, the fluorescence intensity of the SWCNT-miDNA208a sensor gradually increases (Figure S5) up to 125% for the target miRNA and 11% for the control miRNA, respectively (Figure 3e,f). The increased response of the sensor in serum can be due to nonspecific binding of analytes to the SWCNT themselves and not due to hybridization with the DNA.

The SWCNT sample used in this study contains a mixture of different chiralities of SWCNTs. Due to the specific diameter and structure, each chirality differs in the surface configuration of its DNA corona; thus, the fluorescence response toward an analyte can be different for different chiralities.^30,68−73^ However, in the case of the SWCNT-miDNA208a, we observe comparable binding affinities for all chiralities in the range of 0.1–1.8 μM for miRNA and 1.9–2.5 μM for miDNA. The limit of detection (LOD) is in the range of 9–81 nM for the RNA target sequence. Furthermore, we observe the 125% intensity increase in the fluorescence response and the lowest LOD for the (9,4)-chirality around 9 nM (Figure S6, Tables S1 and S2).

In order to improve the LOD, we tested the SWCNT-miDNA208a sensor performance at a single-sensor level. The SWCNTs were immobilized on a poly l-lysine (PLL)-treated glass-bottom well, and the fluorescence of the individual sensors was monitored using a 2D NIR-camera following the addition of 10 μM of miRNA208a or miRNA-R20, for 4 h (Figure S7 and Movies S1, S2, and S3). The fluorescence intensity of the individual SWCNT-miDNA208a showed a gradual increase in response to the miRNA208a target, whereas the random sequence did not induce any fluorescence change. While this assay was performed at a target concentration of 10 μM, such a platform could be used to detect much lower concentrations using smaller sample volumes and longer durations to allow for the target molecules to diffuse and interact with the sensors.^48^

### Specificity and Selectivity of SWCNT-miDNA208a to Its miRNA Target Sequence

**Figure 4:**
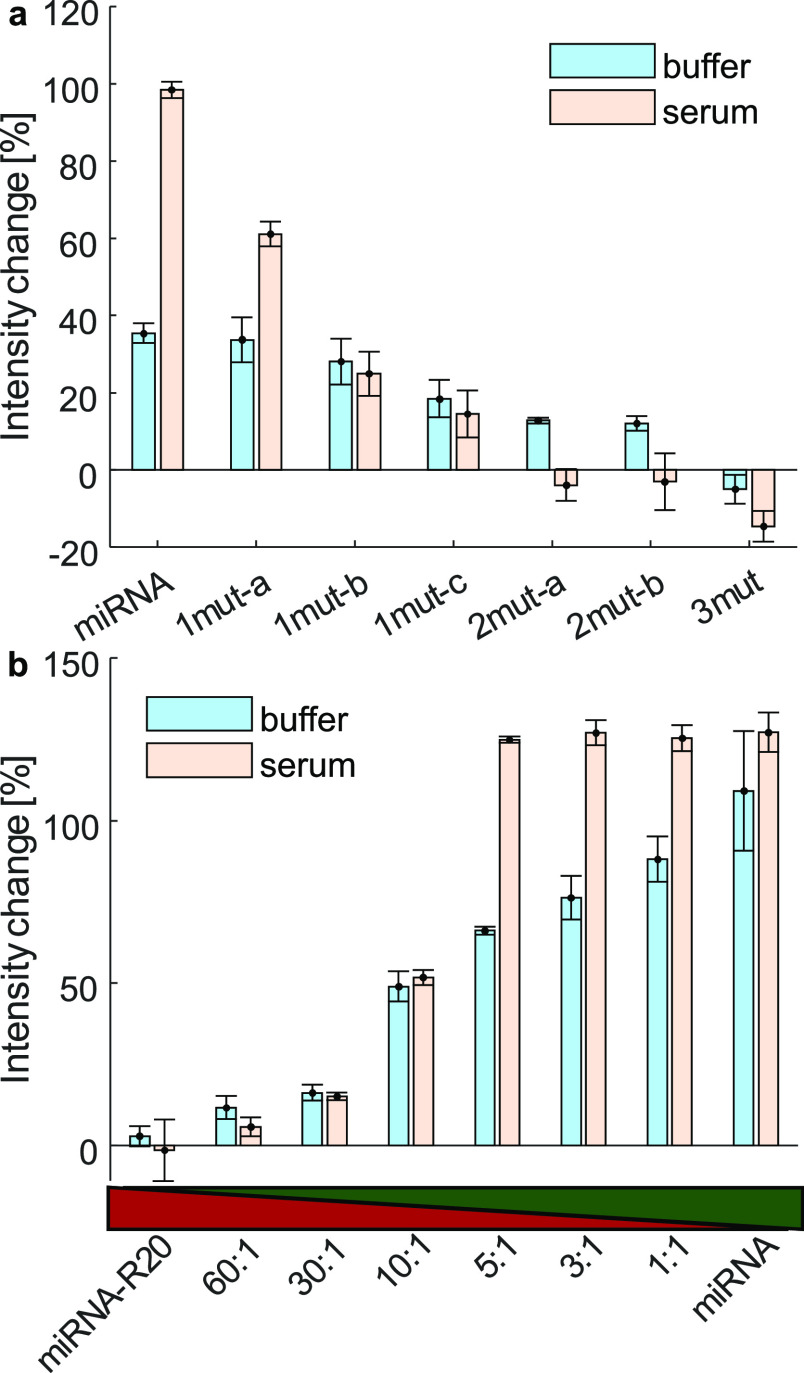
Selectivity and specificity of the SWCNT-miDNA208a sensor. (a) Fluorescence intensity change of SWCNT-miDNA208a following the addition of miRNA208a and different mutated miRNA208a sequences of a single-point mutation, 1mut-a, 1mut-b, 1mut-c; two mutated nucleotides 2mut-a, 2mut-b; and three mutated nucleotides, 3mut. Blue: in buffer, brown: in serum. (b) Fluorescence intensity changes of SWCNT-miDNA208 following the addition of miRNA208a and miRNA-R20 in different ratios. The bar under the graph shows the ratio between miRNA-R20 (dark red) to miRNA208a (dark green). Blue: in buffer, brown: in serum.

The physiological environment contains many oligonucleotide sequences, which could potentially interact with the SWCNTs. In order to assess the specificity and selectivity of the SWCNT-miDNA208a sensor toward its target sequence, we measured its performance in the presence of miRNA208a with point mutations and in a competitive environment, containing a random sequence of miRNA-R20. For the mutated miRNAs, we chose miRNA208a with one mutation (1mut-a, 1mut-b, and 1mut-c), two mutations (2mut-a and 2mut-b), or with three mutated nucleotide bases (3mut). The fluorescence response of the sensor upon the addition of 10 μM of the target miRNA208a or the respective mutated miRNA was measured in buffer and in serum (Figure 4a). Compared to the intensity increase toward the miRNA208a sequence in serum, a single-point mutation resulted in a much smaller increase in the range of 14–61%. Two or three mutations, however, resulted in a fluorescence intensity decrease, which was observed in the previous experiments for the unspecific interaction with the random miDNA. In a similar experiment in buffer, the fluorescence intensity response to the native sequence is similar to the response to two of the sequences with one mutation (1mut-a and 1mut-b), whereas the intensity increase in response to the third single-mutation sequence (1mut-c) is smaller and similar to the response to the sequences with two mutations (2mut-a and 2mut-b). The interaction with the sequence with three mutations (3mut), however, resulted in an intensity decrease. We observe that in a more complex environment such as serum, the specificity of our sensor to miRNA208a is higher than that in buffer. We, therefore, hypothesize that a more crowded environment suppresses the nonspecific binding of the mutated sequences due to increased competition, where only the target sequence can fully hybridize with the complementary DNA segment on the SWCNT corona.

To affirm the selectivity of our SWCNT-miDNA sensor toward its target miRNA, we evaluated the sensor performance in a miRNA-mixture environment with increasing ratios of random miRNA-R20 to miRNA208a while keeping the total concentration of miRNA constant at 30 μM (Figure 4b). In buffer, the SWCNT-miDNA208a sensor showed a gradually increasing response ranging from 0% for the miRNA-R20 alone, 11% fluorescence intensity increase for 60:1 miRNA-R20/miRNA208a ratio, up to 88% for 1:1 ratio. In serum, however, the sensor showed a higher selectivity as the maximal fluorescence intensity increase of 124% was already observed for 5:1 miRNA-R20/miRNA208a. These results demonstrate the selectivity of the SWCNT-miDNA sensor toward its target miRNA, even in the presence of high ratios of random miRNAs.

## Conclusions

Following an extensive elimination process, we have isolated one SWCNT-miDNA optical sensor, which shows high sensitivity and specificity to the AMI-biomarker miRNA208a in a biological, crowded environment out of five potential miRNA biomarkers. The biomarkers for AMI were chosen as a case study to demonstrate the selection pipeline. In our process, we started with functionalized SWCNTs with ssDNA sequences consisting of two regions, an anchor unit, and a complementary sequence to the target. After demonstrating that all of the five tested ssDNA sequences could suspend SWCNT in an aqueous solution, we found that only three out of the five sensor candidates responded to the target miDNA or miRNA in buffer. These three sensors were then assessed in a serum environment, where only one sensor, SWCNT-miDNA208a, showed a fluorescence response to miDNA and was selected for further testing with the target miRNA in serum. In the presence of proteinase K, the SWCNT-miDNA208a showed a significant fluorescence response to the miRNA208a target, demonstrating its potential to be used as an optical sensor with the NIR signal for this AMI biomarker in a biological environment. Other clinically relevant samples will require appropriate optimization of proteinase K concentration, depending on the amount of RNases present. The selected SWCNT-miDNA208a sensor showed high specificity and selectivity toward its target analyte, as demonstrated in the lower response toward mutated miRNA target sequences and its excellent performance in the presence of high ratios of random miRNA strands. The LOD of the sensor was found to be approximately 10 nM. While previous studies demonstrated that fluorescence response manifested in a peak wavelength shift,^11^ here, we chose to focus on the fluorescence intensity modulation due to the large extent of the response. Although wavelength shift detection benefits from internal calibration, intensity change detection does not rely on high-resolution spectroscopic data, and the response can be monitored using a 2D NIR camera without requiring hyperspectral imaging.

Our elimination method shows that experiments in serum, which mimic more accurately the relevant physiological environment of the target biomarker, are crucial for sensor development in general. We have demonstrated the affinity of our DNA corona phase of the SWCNT to the target miRNA. Although the serum environment is more complex and crowded than plain buffer, we found that the SWCNT-miDNA208a selectivity and specificity were higher in serum. Moreover, the fluorescence intensity modulation in response to the target increased dramatically by 3–5 fold in serum compared to buffer, indicating that the complex environment could be beneficial for the sensor performance.

In summary, the SWCNT-miDNA208a, which was selected following a series of experiments with miDNA and miRNA targets, in buffer and in serum, can detect miRNA and function as a fluorescent nanosensor for the AMI biomarker miRNA208a. The SWCNT-fluorescence sensors emit in the biological transparency window and benefit from the high stability, easy functionalization, and biocompatibility of the SWCNTs.^20,74^ While the physiological concentration of miRNA is within the range of 10 fM to 100 pM,^11^ the LOD of our sensor is in the nM range for bulk measurement in the solution phase. Based on our demonstration of miRNA detection with individual SWCNTs immobilized on a surface, future work will focus on trying to increase the sensitivity of the SWCNT sensor to much lower target concentrations, which can possibly be achieved by single-sensor level detection.^48^ Still, identifying the appropriate surface functionalization of SWCNTs for sensing the target in the relevant environment for the final application through the selection and elimination process we have presented is a crucial step in the sensor-development pipeline. This procedure can be extended to other biomarker libraries and other nanosensor technologies.

## Experimental Methods

### Suspension of SWCNTs with Single-Stranded DNA

1 mg of SWCNTs (HiPCO, NanoIntegris) was suspended with 2 mg of single-stranded DNA (IDT) in SSC (Bio-Prep) buffer. The complete list of DNA sequences used for the suspensions can be found in Table S3. For the suspension, the mixture was bath sonicated for 10 min (Elma P-30H, 80 Hz), followed by two cycles of direct tip sonication for 20 min (QSonica Q125, 3 mm tip, 4 W) in an ice bath. The resulting suspension was centrifuged twice for 90 min at 16,100 rcf (Eppendorf, 5424R) in order to separate the individually suspended SWCNTs from aggregates and impurities. After each centrifugation step, 80% of the supernatant was collected, and the pellet was discarded. The concentration of the SWCNT suspensions was determined spectroscopically with the extinction coefficient^75^ of ε_632nm_ = 0.036 L·mg^–1^·cm^–1^ (Figure 1). The presence of DNA in the corona phase of the nanotubes was confirmed by the characteristic absorption peak of DNA in the UV range^76^ in a sample from which the free miDNA was filtered in a centrifuge filter (Amicon, MWCO 100 kDa). In a previous study, the number of DNA strands bound to the (6,5)-SWCNT surface was found to be in the range of 250–280 for (GT)_15_.^77^

### Suspension of SWCNTs with Sodium Cholate

SWCNTs (HiPCO, NanoIntegris) were suspended with 2 wt % SC (Sigma-Aldrich) via bath sonication (Elma P-30H, 80 Hz for 10 min, room temperature), followed by direct tip sonication (QSonica Q125, 12 W for 30 min, twice) in an ice bath. To remove SWCNT aggregates and impurities, the suspension was ultracentrifuged (OPTIMA XPN-80, 41,300 rpm for 4 h), and the top 80% of the supernatant was collected.

### Absorption Spectroscopy

Absorption spectra of the suspensions were recorded on an ultraviolet–visible–NIR (UV–vis–NIR) spectrophotometer (Shimadzu UV-3600 PLUS).

### Near-Infrared Fluorescence Spectroscopy

The fluorescence spectra were recorded in a 96-well plate mounted on an inverted microscope (Olympus IX73). A single-wavelength CW-laser (730 nm, MDL-MD-730-1.5W, Changchun New Industries) was used for excitation. Fluorescence emission was spectrally resolved using a spectrograph (Spectra Pro HRS-300, Princeton Instruments) with a slit width of 500 μm and a grating (150 g/mm), and the fluorescence intensity spectrum was recorded by an InGaAs-camera (PylonIR, Teledyne Princeton Instruments).

### Hybridization Experiments in Buffer and Serum

Hybridization experiments were performed at a SWCNT sensor concentration of 2 mg L^–1^ diluted in SSC with 0.2% w/v SDBS^11^ (Sigma-Aldrich). Target DNA or RNA was added to a final concentration of 10 μM. The complete list of targeted DNA and RNA used for the hybridization experiments can be found in the Supporting Information (Table S4). The control sequence (miDNA-R20 and miRNA-R20) is a random sequence of the same length as the target sequence and with the same %CG content. Samples were incubated for 4 h at room temperature. Hybridization experiments in serum were conducted in 10% w/v FBS (Biological Industries) with 0.2% w/v SDBS and a final target miDNA concentration of 10 μM. Samples were incubated for 4 h, or diluted with the 10% serum, 0.2% SDBS with 0.5 mg mL^–1^ of proteinase K (Roche) with the final concentration of RNA of 10 μM while shaking, at room temperature. Time trace experiments were carried out with 2 mg L^–1^ sensor concentration in SSC + 0.2% SDBS or 10% FBS + 0.2% SDBS + 0.5 mg mL^–1^ proteinase K with buffer or serum, 10 μM miRNA208a or 10 μM miRNA-R20 for 4 h in the microscope with an interval read of 15 min. Calibration experiments were measured with 0.2 mg L^–1^ sensor concentration after 4 h incubation, with different concentrations of miDNA or miRNA, between 0.001 and 5000 nM. The specificity experiment was carried out in buffer and serum with the tested sequence at a concentration of 10 μM. The selectivity experiment was carried out at a total miRNA concentration of 30 μM in different ratios of miRNA208a and miRNA-R20.

### Single-Sensor Level Detection

The detection experiments were carried out in a μ-slide of 18 wells (ibidi). The slide was first treated with PLL (Sigma-Aldrich) for 10 min and then washed with water. The sensor was diluted in SSC to a final concentration of 0.2 mg L^–1^, incubated in the well for 20 min, and washed with SSC + 0.2% SDBS. The slide was placed on the inverted fluorescence microscope (Olympus IX83) with the 100×, 1.3 NA magnification objective (Plan FL). The SWCNT-fluorescence was excited by a 730 nm CW laser (MDL-MD-730-1.5W, Changchun New Industries). The laser excitation light was directed to the sample with a dichroic mirror (900 nm long-pass, Chroma), and the NIR emission of the SWCNTs was detected after an additional 900 nm long-pass emission filter (Chroma, ET900lp) with an InGaAs-camera (Raptor, Ninox 640 VIS-NIR). The fluorescence is recorded following the addition of the target miRNA208a, or the random control sequence miRNA-R20, at a final concentration of 10 μM. The data were analyzed using ImageJ. The process was performed at least three times for each sequence.

## Supporting Information Available

Normalized fluorescence spectra of the SWCNT-miDNA sensor response to miDNA and miDNA-R20 in buffer, miRNA and miRNA-R20 in buffer, and to miDNA and miDNA-R20 in FBS; fluorescence spectra of the SWCNT-miDNA sensor in response to miRNA over time; chirality-dependent response of SWCNT-miDNA; determination of the *K*_a_ for SWCNT-miDNA208a toward miRNA208a for the different chiralities; LOD calculations; SWCNT-miDNA sensor response to miRNA208a at a single-sensor level; and DNA and RNA sequences used in this study. The movies depict single-sensor level experiment conducted with both target or random sequences (PDF)
Single-sensor level detection of miRNA208a target (AVI)
Single-sensor level detection of miRNA208a target (AVI)
Single-sensor level detection of miRNA-R20 (AVI)

## Supplementary Material

se3c00633_si_001.pdf

se3c00633_si_002.avi

se3c00633_si_003.avi

se3c00633_si_004.avi
