# Supplementary material for: Single-Walled Carbon Nanotube Sensor Selection for the Detection of MicroRNA Biomarkers for Acute Myocardial Infarction as a Case Study
Source: ACS Sens. 2023 Sep 13;8(10):3713–22. doi: 10.1021/acssensors.3c00633 (PMC10616859; doi:10.1021/acssensors.3c00633)
Supplement: Supplementary file 1 — se3c00633_si_001.pdf [file se3c00633_si_001.pdf]

Single-walled carbon nanotube sensors selection for the detection of microRNA biomarkers for acute myocardial infarction as a case study

Adi Hendler-Neumark[a], Verena Wulf[a] and Gili Bisker[a,b,c,d,*]

[a] Department of Biomedical Engineering, Faculty of Engineering, Tel Aviv University, Tel Aviv 6997801, Israel

[b] Center for Physics and Chemistry of Living Systems, Tel-Aviv University, Tel Aviv 6997801, Israel

[c] Center for Nanoscience and Nanotechnology, Tel-Aviv University, Tel Aviv 6997801, Israel

[d] Center for Light-Matter Interaction, Tel-Aviv University, Tel Aviv 6997801, Israel

*E-mail: bisker@tauex.tau.ac.il

<u>Supporting Information</u>

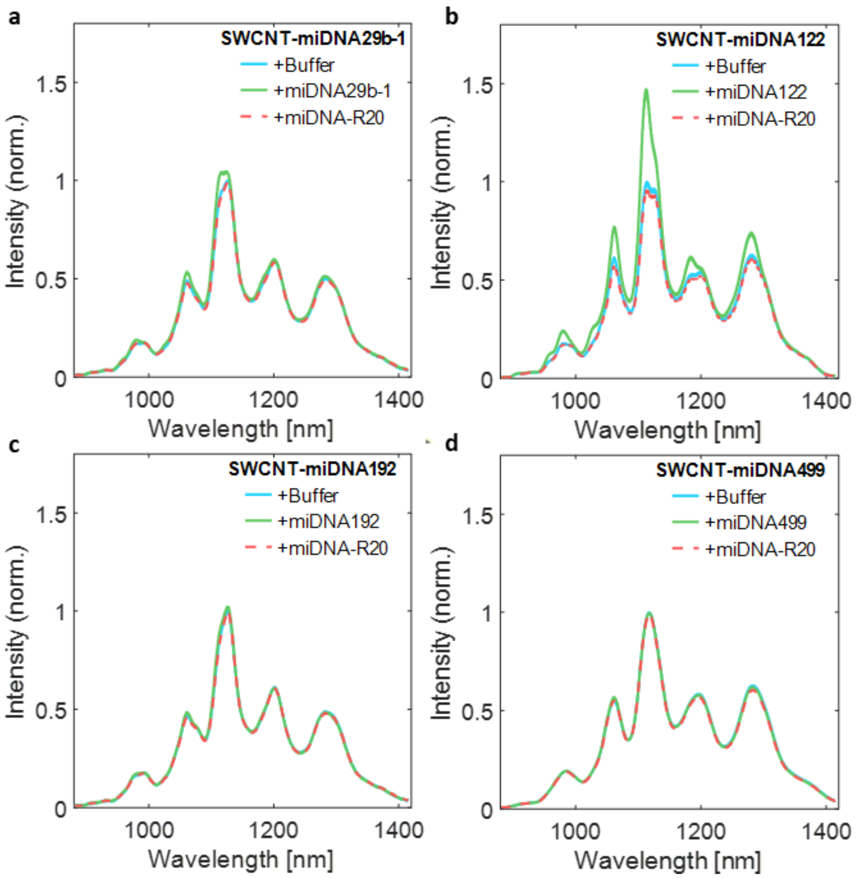

Figure S1: Normalized fluorescence spectra of the SWCNT-miDNA sensor response to miDNA and miDNA-R20 in buffer. a) SWCNT-miDNA29b-1, b) SWCNT-miDNA122, c) SWCNT-miDNA192, d) SWCNT-miDNA499. SWCNT-miDNA sensor in buffer (blue), after the addition of 10 μM miDNA (light green) and after the addition of 10μM miDNA-R20 (light red), following 4 h of incubation.

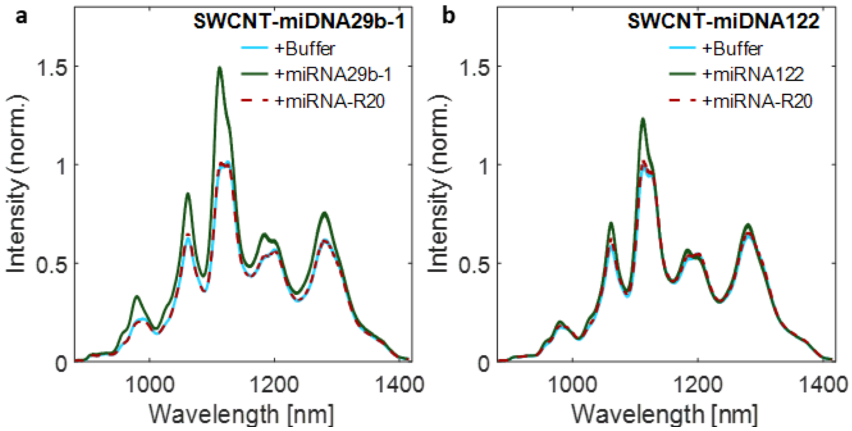

Figure S2: Normalized fluorescence spectra of the SWCNT-miDNA sensor response to miRNA and miRNA-R20 in buffer. a) SWCNT-miDNA29b-1, b) SWCNT-miDNA122. SWCNT-miDNA sensor in buffer (blue), after the addition of 10 µM miRNA (dark green) and after the addition of 10 µM miRNA-R20 (dark red), following 4 h of incubation.

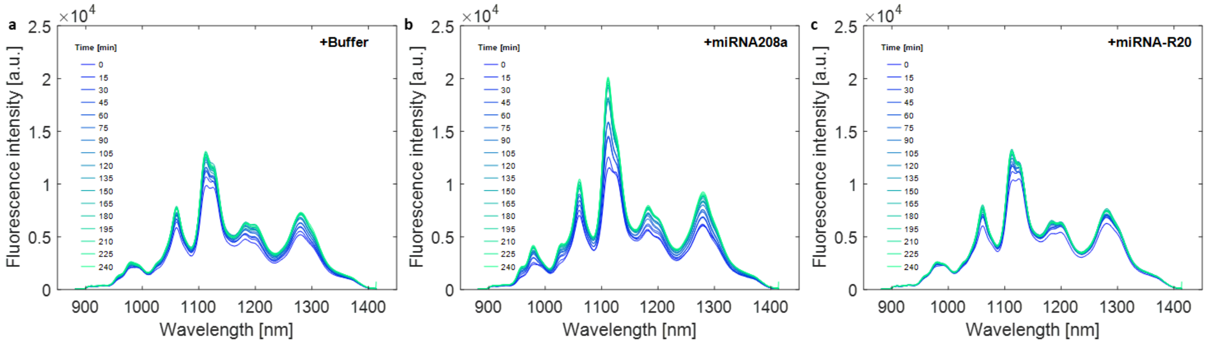

Figure S3: Fluorescence response of the SWCNT-miDNA sensor in buffer over time. Fluorescence spectra of the time-dependent response of the SWCNT-miDNA208a sensor to the addition of (a) buffer, (b) miRNA208a, or (c) miRNA-R20 over 4 h.

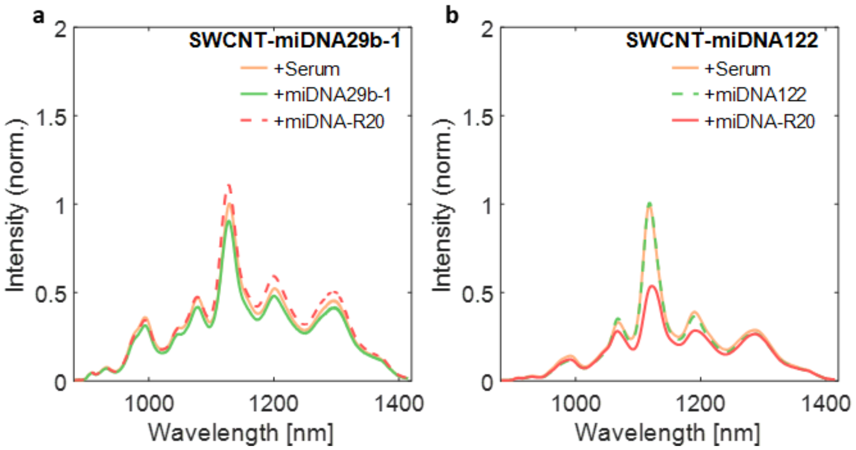

Figure S4: Normalized fluorescence spectra of the SWCNT-miDNA sensor response to miDNA and miDNA-R20 in FBS. a) SWCNT-miDNA29b-1, b) SWCNT-miDNA122. SWCNT-miDNA sensor in serum (brown), after the addition of 10 µM miDNA (light green) and after the addition of 10 µM miDNA-R20 (light red), following 4 h of incubation.

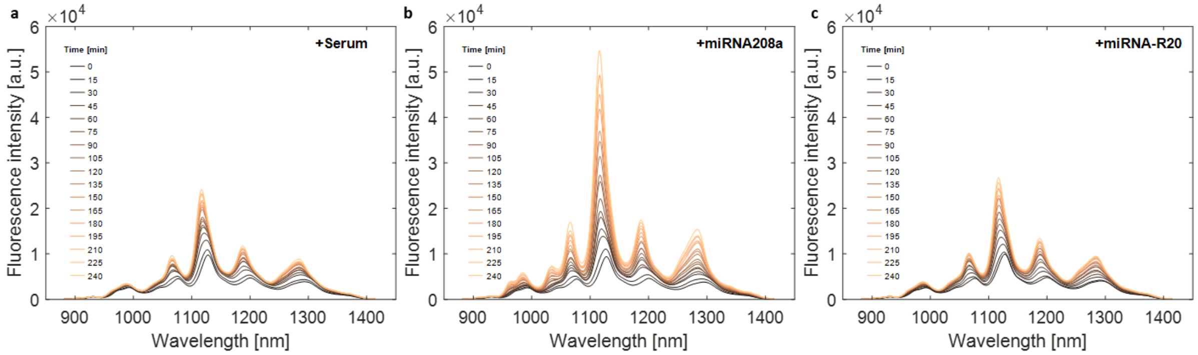

Figure S5: Fluorescence response of the SWCNT-miDNA sensor in serum over time. Fluorescence spectra of the time-dependent response of the SWCNT-miDNA208a sensor to the addition of (a) serum, (b) miRNA208a, or (c) miRNA-R20 over 4 h.

Chirality-dependent response of SWCNT-miDNA208a

To characterize the chirality-dependent response to the target miRNA and miDNA, we measured the concentration-dependent fluorescence response of SWCNT-miDNA208a to miRNA208a or miDNA208a in serum, under an excitation wavelength of λ_{ex}=730 nm. We plotted the normalized fluorescence response of SWCNT-miDNA208a at the peak intensities, corresponding to the (10,2), (9,4), (8,6) and (8,7) chiralities (Figure S6a), resulting in 4 chirality-specific calibration curves, fitted by the Hill isothermal model (Figure S5b and S5c)[1]:

$$\frac{I - I_0}{I_0} = \beta \frac{C_I^n}{K_a^n + C_I^n}$$

where I_0 is the initial fluorescence intensity of the sensor in serum, I is the fluorescence intensity after the addition of the target sequence, β is a proportionality factor, C_I is the concentration of the target miRNA208a or miDNA208a, K_a is the target concentration resulting in 50% of the maximal response and n is the Hill coefficient. The fit parameters for the different chiralities are summed up in Table S1 and Table S2.

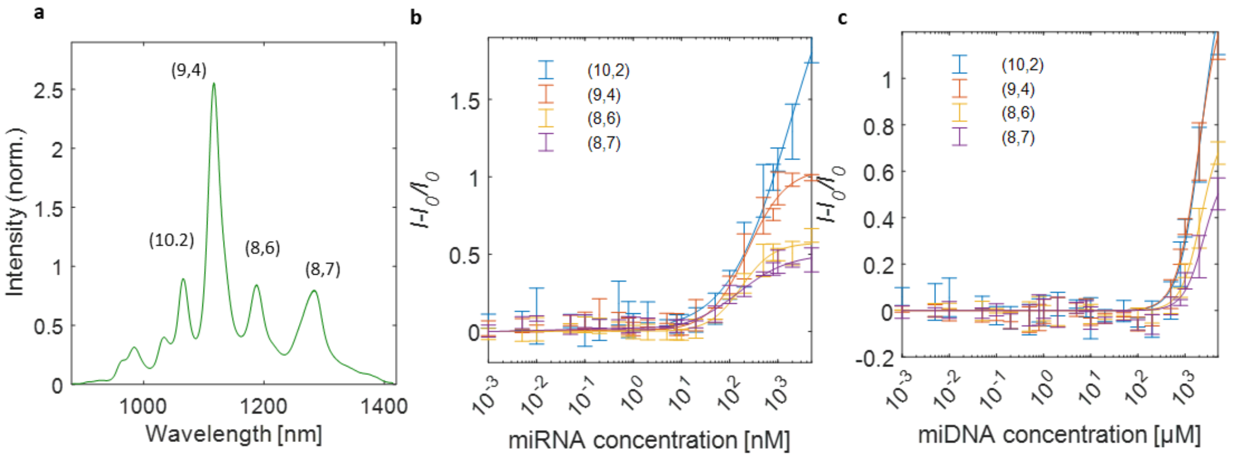

Figure S6: Determination of the K_a for SWCNT-miDNA208a towards miRNA208a or miDNA208a for the different chiralities. a) Fluorescence spectrum of SWCNT-miDNA208a in serum (FBS) captured at an excitation wavelength of λ_{ex} = 730 nm with the different chiralities assigned. b) Concentrations dependent fluorescence response of SWCNT-miDNA208a (dots and error bars) and the corresponding fit according to the Hill isothermal model (lines) for the different chiralities: (10,2)-blue, (9,4)-red, (8,6)-yellow and (8,7)-purple for miRNA208a and c) miDNA208a.

Table S1: Limit of detection (LOD), K_a, proportionality factor (β) and Hill coefficient (n) for sensors in serum with miRNA

	**miRNA208a**			
Chirality	**(10,2)**	**(9,4)**	**(8,6)**	**(8,7)**
LOD (nM)	81.09	9.01	28.09	36.7
K_a (µM)	1.8±2.2	0.2±0.9	0.1±0.05	0.15±0.12
β	2.7±1.04	1.05±0.12	0.57±0.55	0.5±0.1
n	0.66±0.18	1.08±0.3	1.3±0.37	0.82±0.33

Table S2: Limit of detection (LOD), K_a, proportionality factor (β) and Hill coefficient (n) for sensors in serum with miDNA

	**miDNA208a**			
Chirality	**(10,2)**	**(9,4)**	**(8,6)**	**(8,7)**
LOD (nM)	492.6	323.8	633.9	819.7
K_a (µM)	2.2±0.6	1.9±0.4	2.1±0.6	2.5±1.5
β	1.54±0.3	1.38±0.2	0.8±0.1	0.62±0.2
n	1.8±0.5	1.86±0.4	2.03±0.58	2.01±1.1

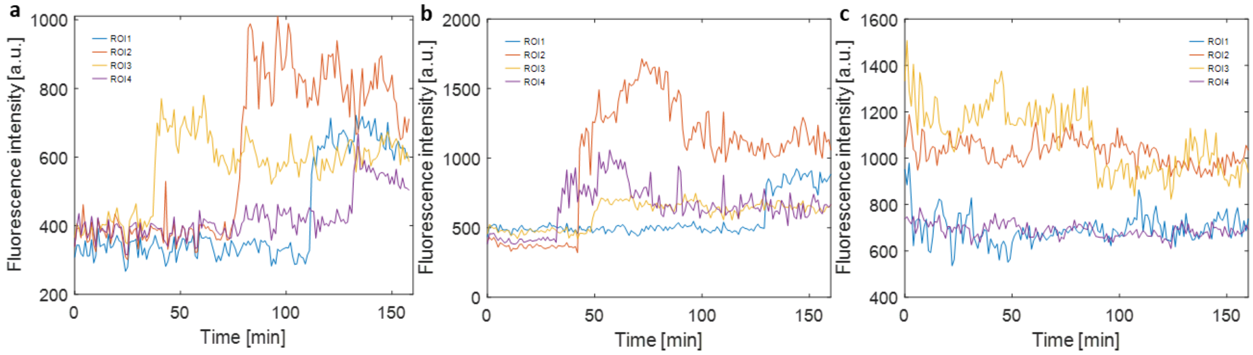

Figure S7: Single-sensor level detection. The fluorescence intensity of individual SWCNT-miDNA208a immobilized on a surface using a 2D NIR camera. (a), (b) The fluorescence of 4 different ROIs with single sensors in blue, red, yellow, and purple, after the addition of 10 µM of miRNA208a from Movies S1 and S2 with the interval of 1 min. (c) The fluorescence intensity of 4 different ROIs with single sensors in blue, red, yellow, and purple after the addition of 10 µM of miRNA-R20 from Movie S3 with the interval of 1 min.

Table S3: DNA for SWCNT functionalization. The underlined sequence in red is the complementary sequence to the target miDNA and miRNA.

**Name**	**Sequence**
miDNA122-sensor	GTGTGTGTGTGTGTGTGTGTGTGTGTGTGTAAATCACACTATTACCGCAA
midNA499a-sensor	GTGTGTGTGTGTGTGTGTGTGTGTGTGTGTCGTGTCTGAACGACACTACA
midNA192-sensor	GTGTGTGTGTGTGTGTGTGTGTGTGTGTGTACTGGATACCTTAACCGTA
midNA29b-1-sensor	GTGTGTGTGTGTGTGTGTGTGTGTGTGTGTTGACTAAAGTTTACCACGAT
miDNA208a-sensor	GTGTGTGTGTGTGTGTGTGTGTGTGTGTGTTTCGAAAAACGAGCAGAATA

Table S4: DNA and RNA target sequences

**Name**	**Sequence**
miDNA122	TTGCGGTAATAGTGTGATTT
miRNA122	UUGCGGUAAUAGUGUGAUUU
miDNA499	TTGTAGTGTCGTTCAGACACG
miDNA192	TACGGTTAAGGTATCCAGT
miDNA29b-1	ATCGTGGTAAACTTTAGTCA
miRNA29b-1	AUCGUGGUAAACUUUAGUCA
miDNA208a	TATTCTGCTCGTTTTTCGAA
miRNA208a	UAUUCUGCUCGUUUUUCGAA
miRNA208a-1mut-a	UAUACUGCUCGUUUUUCGAA
miRNA208a-1mut-b	UAUUCUGCACGUUUUUCGAA
miRNA208a-1mut-c	UAUUCUGCUCGUUUAUCGAA
miRNA208a-2mut-a	UAUACUGCUCGUUUAUCGAA
miRNA208a-2mut-b	UAUACUGCACGUUUUUCGAA
miRNA208a-3mut	UAUACUGCACGUUUAUCGAA
miDNA-R20	ATCCGTATTTGATGCTACCA
miRNA-R20	AUCCGUAUUUGAUGCUACCA

Movies:

Movie S1 and S2: Single sensor level detection of miRNA208a target. The ROIs are marked with the same color as the corresponding data in Figure S6a and S6b (blue, red, yellow, and purple).

Movie S3: Single sensor level detection of miRNA-R20. The ROIs are marked by the same color as the corresponding data in Figure S6c (blue, red, yellow, and purple).

References

(1) Foo, K. Y.; Hameed, B. H. Insights into the Modeling of Adsorption Isotherm Systems. *Chem. Eng. J.* **2010**, *156*, 2–10.
